# Cognitive and adaptive pathways linking self-efficacy and job crafting: the moderating role of perceived organizational support among female knowledge workers in Chinese tourism enterprises

**DOI:** 10.3389/fpubh.2026.1886029

**Published:** 2026-08-12

**Authors:** Zhuquan Yang, Xiaoxin Xie

**Affiliations:** 1 University Engineering Research Center of Full-Chain Dynamic Innovation in Wellness Tourism, Hezhou University, Hezhou, China; 2 School of Tourism and Sport Health, Hezhou University, Hezhou, China

**Keywords:** conservation of resources theory, female knowledge workers, job crafting, perceived organizational support, psychological resilience, self-efficacy, self-reflection, tourism enterprises

## Abstract

**Background:**

Female knowledge workers in tourism enterprises face intensive emotional labor, seasonal demand fluctuation, and gendered work-family expectations that shape their capacity for proactive job redesign. However, the psychological mechanisms through which self-efficacy is associated with job crafting in this population, and the boundary conditions under which contextual resources amplify or substitute for internal resources, remain underspecified.

**Methods:**

Drawing on Conservation of Resources (COR) theory, this study investigates how self-efficacy is associated with job crafting through parallel mediation by self-reflection and psychological resilience, and how perceived organizational support (POS) conditions these pathways. Survey data were collected from 576 female employees in Chinese tourism enterprises across four provinces. Analyses included confirmatory factor analysis, hierarchical regression, and bootstrap-based moderated mediation.

**Results:**

Self-efficacy shows a significant positive association with job crafting (β = 0.298, *p* < 0.001). Self-reflection and psychological resilience each partially mediate this association, with the psychological resilience pathway (β = 0.354) approximately 2.5 times stronger than the self-reflection pathway (β = 0.141). POS positively moderates the self-reflection–job crafting association (β = 0.077, *p* = 0.021), but the corresponding moderated mediation is not statistically significant. Contrary to prediction, POS significantly weakens the psychological resilience–job crafting relationship (β = −0.128, *p* = 0.001), and the moderated mediation index is negative and significant [Index = −0.101, 95% CI (−0.174, −0.016)], revealing a resource substitution effect.

**Conclusions:**

This study contributes to the job crafting literature by distinguishing cognitive and adaptive resource pathways through which self-efficacy operates, and by demonstrating that organizational support functions asymmetrically across pathways—amplifying cognitive resource conversion while substituting for adaptive resource conversion. Tourism enterprises should balance organizational support with employees' internal resource development.

## 1 Introduction

In increasingly dynamic and uncertain work environments, employees are no longer passive recipients of pre-defined job roles but active agents who continuously adjust their work to maintain effectiveness and wellbeing. This tendency is especially pronounced in service-intensive industries such as tourism and hospitality, where emotional labor, fluctuating customer demands, seasonal workload variation, and situational unpredictability require employees to proactively adapt how they perform and interpret their work (1). Job crafting—employees' self-initiated modifications of task boundaries, social interactions, and cognitive perceptions—has therefore become a central concept in organizational behavior and human resource management research (2). Prior studies have demonstrated that job crafting is associated with higher work engagement, improved performance, and sustainable career development (3, 4).

Existing research has identified a range of antecedents associated with job crafting. At the individual level, personal characteristics such as proactive personality, motivation, and self-efficacy are consistently linked to higher levels of job crafting (3, 5). Among these factors, self-efficacy has been recognized as a particularly important psychological resource, as employees who believe in their ability to handle work demands are more likely to take initiative and reshape their jobs (6, 7). At the contextual level, leadership styles, organizational climate, and perceived organizational support have also been shown to influence job crafting by reducing perceived risks and enhancing psychological safety (8, 9). However, three specific and interrelated gaps in this literature motivate the present study.

First, although self-efficacy is widely recognized as a correlate of job crafting, the psychological mechanisms through which this conversion occurs remain underspecified. Most existing studies conceptualize the self-efficacy–job crafting relationship as either a direct effect or a single-mediator process, typically involving work engagement or motivation (7, 10). Yet cognitive processing and adaptive functioning are theoretically distinct capacities, and it is unlikely that a single mediator can capture how efficacy beliefs relate to proactive behavior in complex service environments. Specifically, prior research has not examined whether cognitive resources (such as self-reflection) and adaptive resources (such as psychological resilience) operate as parallel mechanisms through which self-efficacy is converted into job crafting. This gap limits our theoretical understanding of the multi-pathway nature of resource conversion.

Second, although Conservation of Resources (COR) theory emphasizes the interaction between personal and contextual resources (11, 12), perceived organizational support (POS) has been pre-dominantly modeled as a uniformly positive moderator that amplifies personal-resource effects on proactive behavior (13, 14). This framing overlooks a key theoretical possibility embedded within COR—namely, that abundant contextual resources may function not only as an amplifier of internal resources but also as a substitute for them, particularly when internal resources and external resources address overlapping functional demands (15). To date, empirical research has rarely tested whether POS may show asymmetric associations across different psychological pathways.

Third, female knowledge workers in Chinese tourism enterprises constitute a workforce whose distinctive occupational conditions have received limited scholarly attention. Women constitute the numerical majority in China's tourism industry (16), concentrated in customer-facing and coordination-intensive roles such as front-desk reception, ticketing management, tour operations, and cultural product design. These roles are characterized by intensive emotional labor, seasonal demand fluctuation, service unpredictability, career uncertainty, and gendered work-family expectations that require simultaneous negotiation of professional and domestic responsibilities (17, 18). Existing job crafting research has been conducted pre-dominantly on mixed-gender or Western samples, and specific psychological mechanisms through which self-efficacy is associated with job crafting under these gendered service-intensive conditions have not been systematically examined.

To address these three gaps, this study adopts Conservation of Resources (COR) theory as its primary theoretical framework. COR provides an integrated resource-conversion logic that explains how personal resources are strategically mobilized, how they interact with contextual resources, and how resource investment is shaped by boundary conditions. Social Cognitive Theory (SCT) is drawn on in a supporting role, specifically to justify the conceptualization of self-efficacy as a core capability belief that motivates resource investment (19); the mediating mechanisms, moderating logic, and pathway-specific interactions are all articulated within the COR framework. From this integrated perspective, self-efficacy is conceptualized as a core personal resource that motivates job crafting (11), operating through two parallel psychological pathways: a cognitive pathway represented by self-reflection, which may support sense-making and strategic adjustment (20), and an adaptive pathway represented by psychological resilience, which supports sustained functioning under pressure and change (21, 22). Perceived organizational support is conceptualized as a conditional contextual resource that may either amplify or substitute for these internal pathways (12, 15).

Focusing on female knowledge workers in Chinese tourism enterprises, this study addresses the following research questions: (RQ1) How does self-efficacy influence job crafting among female knowledge workers in tourism enterprises? (RQ2) Do self-reflection and psychological resilience function as parallel psychological mechanisms through which self-efficacy is transformed into job crafting? (RQ3) How does perceived organizational support condition the effects of self-reflection and psychological resilience on job crafting, and are these boundary effects symmetric or asymmetric across pathways?

By answering these questions, this study makes three contributions. Theoretically, it advances job crafting research by distinguishing cognitive and adaptive resource pathways through which self-efficacy operates and it extends COR theory by demonstrating that contextual resources may show asymmetric associations—amplifying some psychological pathways while substituting for others. Contextually, it extends job crafting research to female knowledge workers in Chinese tourism enterprises, a population whose occupational conditions demand focused theoretical attention. Practically, the findings offer guidance for tourism enterprises on how to balance organizational support with employees' internal resource development.

## 2 Theoretical basis and research hypotheses

### 2.1 Theoretical basis

This study adopts Conservation of Resources (COR) theory (11, 12) as its primary theoretical framework. COR posits that individuals strive to acquire, retain, and protect resources that they value, and that resource investment—the mobilization of existing resources to attain further resources—constitutes a central mechanism through which proactive behaviors emerge. From this perspective, personal resources such as self-efficacy and psychological resilience do not automatically be associated with proactive work behaviors. Rather, the conversion of internal resources into action involves non-negligible costs. Moving from a psychological state of feeling competent and resilient to the actual practice of job crafting requires employees to invest time and energy, tolerate uncertainty, and accept the risk of failure (7, 10).

Social Cognitive Theory (SCT) is drawn on in a supporting capacity to justify the specific status of self-efficacy in this framework. SCT conceptualizes self-efficacy as a domain-general capability belief that shapes goal-setting, effort allocation, and persistence (19). Within our COR-based model, SCT provides theoretical grounding for treating self-efficacy as a core personal resource that motivates resource investment, while the mediating pathways, moderating logic, and pathway-specific resource interactions are all articulated in COR terms.

Prior studies on job crafting emphasize that engaging in job crafting requires specific cognitive and behavioral capacities, such as reflecting on past work experiences, identifying opportunities for change, and proactively implementing adjustments without formal authority (1, 4). These skill requirements imply that even employees with abundant personal resources may refrain from job crafting when the perceived cost of action outweighs the anticipated resource gains (3). Accordingly, COR suggests that personal resources facilitate proactive behaviors by reducing the perceived cost of action rather than eliminating it entirely (12). Employees with higher self-efficacy are more likely to perceive the investment of effort and time as manageable and worthwhile, while psychological resilience enables individuals to persist in the face of potential setbacks during the job crafting process (6, 23). In this sense, job crafting represents a resource investment process in which personal resources are strategically mobilized to offset conversion costs and increase the likelihood of proactive action. Importantly, COR further recognizes that contextual resources—such as perceived organizational support—may either amplify the effects of personal resources by supplying complementary support, or substitute for them when contextual resources fulfill overlapping functional demands (15). This dual role of contextual resources provides the theoretical basis for examining POS as an asymmetric moderator in the present study.

### 2.2 Research hypotheses

#### 2.2.1 The relationship between self-efficacy and job crafting

Self-efficacy refers to an individual's belief in their ability to complete specific tasks or cope with particular situations. Grounded in Bandura's Social Cognitive Theory (19) and further articulated in COR theory as a core personal resource (11), self-efficacy drives employees to actively shape their work content, methods, and relationships (6). In dynamic and uncertain environments such as tourism enterprises, self-efficacy is especially critical. It is associated with employees identifying and seizing opportunities for role redesign in the face of fluctuating customer demands, emotional labor, and service unpredictability (24). Research has shown that employees with higher levels of self-efficacy are more likely to proactively adjust their work tasks and interaction patterns to enhance their sense of personal value and person–job fit (25). When faced with work challenges or resource shortages, high self-efficacy helps employees maintain a positive attitude and confidence in problem-solving, thereby motivating them to engage in job crafting (26). In tourism enterprises that emphasize service quality and customer experience, self-efficacy therefore influences both employees' individual work motivation and the organization's overall service innovation capacity (27).

Empirical studies provide substantial evidence supporting a significant positive relationship between self-efficacy and job crafting. The Job Crafting Self-Efficacy Scale developed by Roczniewska et al. (6) shows that employees with higher scores are more proactive in task reconstruction, relational restructuring, and cognitive reframing. Pelit et al. (28) found that tour guides' self-efficacy was significantly positively correlated with their task expansion and service innovation behaviors. Guo et al. (25) further revealed that customer empowerment behaviors could indirectly be associated with job crafting by enhancing employees' self-efficacy. Hur and Moon (26) noted that, in the context of the pandemic, service workers with higher self-efficacy were able to adjust their work methods more quickly and maintain performance. Ahmed et al. (27) also found that self-efficacy facilitated more proactive work innovation behaviors among hotel industry employees. Based on the theoretical and empirical foundation outlined above, this study proposes:

**H1:** Self-efficacy is positively associated with female knowledge workers' job crafting in tourism enterprises.

#### 2.2.2 The mediating role of self-reflection

Self-reflection refers to the metacognitive process in which individuals review, assess, and reconstruct their own behaviors, situational judgments, and outcomes. It enhances self-awareness, corrective learning, and situational adaptability through a “reflection–learning–action” cycle (29). Although much of the empirical literature on self-reflection originates in educational and clinical contexts, the underlying cognitive mechanism—the metacognitive processing of experience into revised action—is theorized as a domain-general capability rather than a context-specific one (29, 30). This domain-generality is particularly relevant to tourism enterprise work, which shares three structural features with educational and clinical service work: (a) high-frequency interpersonal service encounters that require ongoing interpretation of others' responses; (b) emotionally demanding interactions that necessitate deliberate cognitive processing to sustain quality; and (c) fluctuating situational demands that reward experience-based adjustment. Prior evidence from tourism and hospitality research has confirmed that reflective cognition operates similarly in service-intensive contexts (31, 32). On this basis, we draw on cross-context evidence to develop the mediating hypothesis, while grounding the specific mechanism in the tourism enterprise context.

In service-intensive and fast-changing tourism enterprises, critical and constructive reflection is regarded as a crucial mechanism for maintaining professional competence and service quality (31). Research in professional practice has shown that systematic reflection can promote learners' self-efficacy and professional competence, forming a resource “gain spiral” that aligns with the resource accumulation and reinvestment propositions in COR theory (33). Self-reflection thus functions as both a cognitive personal resource and a key “converter” linking experience with improvement actions, providing the psychological and cognitive preparation for subsequent proactive behaviors such as job crafting.

A significant body of recent research shows a positive relationship between self-efficacy and self-reflection. In educational and professional settings, individuals with higher self-efficacy are more likely to engage in metacognitive and reflective activities involving planning, monitoring, and evaluation, which in turn solidify their abilities and motivation (34, 35). Studies involving health and nursing samples also suggest that self-reflection and self-efficacy enhance each other, indicating a positive correlation between the two (30). In curriculum and training design research, embedding reflection into learning units has been shown to significantly enhance learners' self-efficacy, providing indirect support for the “efficacy–reflection–efficacy” resource gain cycle (33). In tourism and hospitality industries facing digitalization, cross-cultural challenges, and AI empowerment, female knowledge workers with higher self-efficacy are similarly more likely to engage in situational review and strategic examination to maintain control and growth in high-uncertainty situations—consistent with COR's “resources promote resources” mechanism.

In organizational and service research, reflection has been shown to improve self-insight and the clarity of needs and values, thereby motivating employees to proactively adjust their task, relational, and cognitive boundaries—that is, to engage in job crafting (36). From a mechanistic perspective, reflection may support meaning construction and cognitive reframing, thereby enhancing the tendency to reshape tasks and relationships (4). In tourism and hospitality contexts, frontline employees facing customer mistreatment, fluctuating demands, and complex processes engage in an “avoidance–optimization–innovation” strategy of job crafting through reflection and evaluation (32). Additionally, after receiving customer empowerment and situational feedback, service providers often translate this into specific job crafting behaviors via the pathway of self-efficacy and reflection (37). When self-efficacy increases, employees are therefore more likely to engage in self-reflection and subsequently implement job crafting, with reflection playing a bridging role in cognitive transformation and action implementation—consistent with COR's assertion that personal resources are transformed into resource investment behaviors through cognitive processing (4, 36, 37).

**H2**: Self-reflection mediates the relationship between self-efficacy and job crafting.

#### 2.2.3 The mediating role of psychological resilience

Psychological resilience refers to an individual's capacity to maintain positive functioning, adapt effectively, and recover from setbacks when facing adversity, stress, and change (38). As a general adaptive resource, psychological resilience encompasses emotional stability, stress management, positive appraisal of challenges, and behavioral perseverance under pressure (38). Within COR theory, psychological resilience is conceptualized as a core personal resource that enables individuals to buffer resource loss and mobilize resource reinvestment when facing demanding conditions (11, 12). In service-intensive work environments characterized by high emotional labor, unpredictable customer interactions, and cyclical workload pressure, psychological resilience is particularly relevant as it allows employees to sustain proactive functioning rather than withdrawing under strain (21, 22).

Social Cognitive Theory and positive psychology suggest that self-efficacy is an important antecedent of psychological resilience because it directly influences an individual's appraisal of and response strategies to challenging situations (19). Employees with high self-efficacy are more willing to proactively confront demanding situations, learn from setbacks, and mobilize adaptive strategies, all of which reinforce their psychological resilience (34). In the tourism industry, this dynamic is intensified: employees with high self-efficacy are more willing to proactively learn new skills, adjust their responses to peak-period pressures, and adapt to fluctuating customer demands, thereby sustaining psychological resilience under conditions of high emotional labor and situational uncertainty (21, 22). Research indicates that self-efficacy not only boosts employees' confidence in coping but also indirectly enhances their psychological resilience by stimulating proactive coping strategies and increasing stress tolerance (22).

Job Crafting Theory emphasizes that individuals actively adjust task boundaries, interpersonal interactions, and cognitive frameworks to improve their work experience and performance (1). Psychological resilience, as a dynamic adaptation capability, enhances individuals' psychological sense of safety and resource mobilization ability when faced with job changes, thereby supporting job crafting behavior (39). Resilient individuals are more capable of tolerating the uncertainty inherent in proactive role redesign, sustaining effort through initial setbacks, and reinterpreting challenges as opportunities for growth. In the tourism industry, employees with high psychological resilience are more likely to identify improvement opportunities in service processes, proactively take on new tasks, and adjust service approaches to enhance both customer experience and their own sense of accomplishment (40). Based on COR theory, psychological resilience not only helps individuals buffer resource loss but also may facilitate the resource gain spiral, thus playing a key mediating role between self-efficacy and job crafting (41).

**H3**: Psychological resilience mediates the relationship between self-efficacy and job crafting.

#### 2.2.4 The moderating role of perceived organizational support between self-reflection and job crafting

Perceived organizational support (POS) refers to the extent to which employees perceive that the organization values, cares for, and recognizes their contributions at both the material and emotional levels (42). High levels of POS enhance employees' emotional commitment, organizational identification, and psychological safety, thereby motivating them to engage in more proactive work behaviors (43). In service-intensive contexts such as tourism and hospitality, POS also manifests as the organization's tolerance and support for employees' creative service delivery and flexible responses to customer demands, providing psychological buffering when employees face uncertainty and high customer-contact environments (44). Social Exchange Theory suggests that when employees feel supported by the organization, they are more likely to reciprocate with positive work behaviors (14, 45), providing a solid theoretical foundation for studying POS as a moderating variable.

Within the cognitive pathway to job crafting, POS is expected to amplify the conversion of self-reflection into concrete job crafting behavior. Self-reflection generates cognitive resources—insight, meaning construction, and identification of improvement opportunities—but the translation of these cognitive resources into behavioral action requires an environment in which experimentation, initiative, and boundary adjustment are supported rather than penalized (42, 46). When employees perceive high organizational support, they are more likely to trust that reflection-derived insights can be safely enacted, more willing to invest effort in behavioral change, and more confident that potential risks associated with proactive role redesign will be organizationally absorbed (46, 47). In this sense, POS provides the enabling contextual resource that closes the gap between cognitive processing and behavioral implementation. From a COR perspective, POS complements the cognitive resource of self-reflection by supplying safety, sanction, and material affordances necessary for resource investment (12, 15). Cheng and O-Yang (46) found in the hotel industry that POS significantly enhances the extent to which employees translate cognitive processing into task crafting, relational crafting, and cognitive crafting, precisely because the availability of organizational resources reduces the risks associated with innovation attempts. Uçar and Kerse (47) similarly showed that POS is not only directly associated with job crafting but also increases employees' acceptance of new processes.

**H4**: Perceived organizational support positively moderates the relationship between self-reflection and job crafting, such that the effect of self-reflection on job crafting is stronger when POS is higher.

#### 2.2.5 The moderating role of perceived organizational support between psychological resilience and job crafting

Empirical research has generally found that POS plays an important moderating role in the relationship between individual psychological resources and proactive work behaviors. In high organizational health climate contexts, employees' resilience has been found to more strongly be associated with job crafting behaviors (43). In the hotel industry, POS has been shown to moderate the relationship between job crafting and employee satisfaction and burnout: in high-POS environments, job crafting behaviors are more likely to enhance satisfaction and reduce burnout (46). Ok and Lim (48) pointed out that in environments with high POS, employees are more likely to achieve better person–job fit through task and cognitive crafting, which leads to higher innovative behaviors. These findings suggest that in tourism-related contexts, POS may help translate psychological resilience into specific job crafting actions.

According to COR theory, contextual resources such as POS can facilitate the transformation of personal psychological resources such as resilience into behavioral outcomes such as job crafting, creating a resource gain spiral (43). The above empirical studies support a mechanism in which POS enhances the strength of the resilience-to-job-crafting path. In tourism enterprises, female knowledge workers in high-POS environments would thus be expected to convert their psychological resilience into proactive behaviors of adjusting work tasks, relationships, and cognition more effectively than those in low-POS environments. Based on this line of reasoning:

**H5**: Perceived organizational support positively moderates the relationship between psychological resilience and job crafting, such that the effect of psychological resilience on job crafting is stronger when POS is higher.

#### 2.2.6 The moderated mediation model

**Figure 1:**
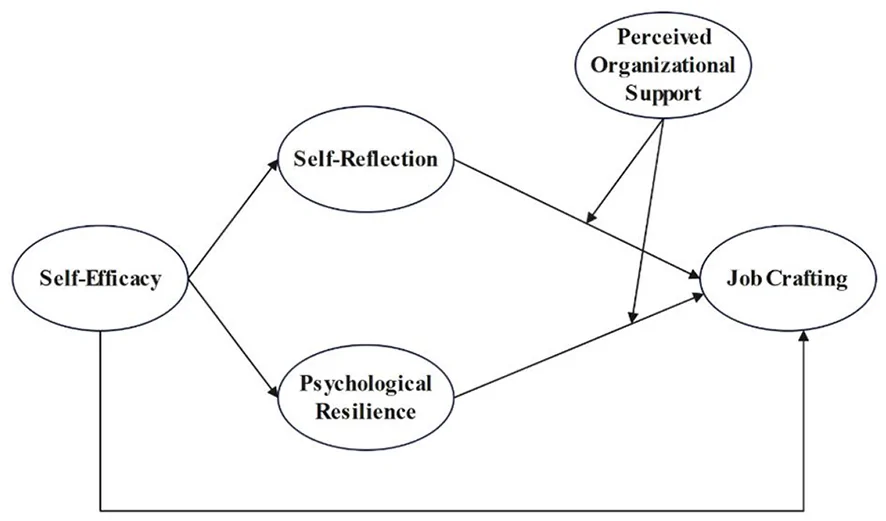
Theoretical framework model. SE, self-efficacy; SR, self-reflection; PR, psychological resilience; POS, perceived organizational support; JC, job crafting.

In summary, this study constructs a moderated mediation model (see Figure 1) that integrates the effects of self-efficacy, self-reflection, psychological resilience, and perceived organizational support on job crafting. Self-efficacy is theorized to influence job crafting through two parallel psychological pathways—a cognitive pathway via self-reflection and an adaptive pathway via psychological resilience—with POS moderating both mediated pathways.

## 3 Materials and methods

### 3.1 Measurement instruments

#### 3.1.1 Self-efficacy scale

Self-efficacy was measured using a 12-item scale contextually adapted from Tschannen-Moran and Hoy (49). The theoretical grounding of this measure lies in Bandura's (19) domain-general conceptualization of self-efficacy as beliefs about one's capability to organize and execute the courses of action required to attain designated outcomes. Bandura explicitly argued that self-efficacy is not tied to any specific occupational context, but that its measurement must be operationalized through context-relevant behaviors. Tschannen-Moran and Hoy (49) themselves positioned their instrument as a Bandurian operationalization for the teaching context rather than as a construct independent of the general self-efficacy framework, and the three underlying functional dimensions of their original scale—efficacy for management, efficacy for motivation, and efficacy for cooperation—represent generalizable workplace capability domains rather than teaching-exclusive competencies.

These three capability domains map directly onto the core work demands of female knowledge workers in tourism enterprises, whose daily responsibilities involve: (a) managing dynamic and often unpredictable service situations such as customer complaints, service disruptions, and cross-departmental coordination; (b) motivating team members and sustaining customer engagement under conditions of emotional labor and demand fluctuation; and (c) maintaining cooperative interactions across multiple service touchpoints. Given this structural correspondence, we systematically adapted the original items to the tourism enterprise context during the standard translation and back-translation procedure (50, 51). Specifically, educational referents in the original items (e.g., “students,” “classroom,” “instructional strategies”) were replaced with functionally equivalent tourism work referents (e.g., “colleagues/customers,” “work,” “work strategies”) while preserving the capability structure of each item. For example, the original item “How much can you do to control disruptive behavior in the classroom?” was adapted to “How capable are you of handling disruptive incidents at work?,” and “How much can you do to motivate students who show low interest in schoolwork?” was adapted to “How capable are you of motivating colleagues who show low engagement in their work?.” The complete adapted item list is provided in Supplementary Table S1.

The adapted scale demonstrated strong psychometric properties in the present sample (see Section 4.3 and Table 1): all 12 standardized factor loadings ranged from 0.667 to 0.861, with AVE = 0.653, CR = 0.957, and Cronbach's α = 0.959. These indicators substantially exceed conventional thresholds (52, 53) and provide empirical evidence that the adapted scale captures a coherent self-efficacy construct in this population.

**Table 1:** Reliability and convergent validity.

Factor	Item	Std. estimate	AVE	CR	Cronbach's α
Self-efficacy	SE1	0.667	0.653	0.957	0.959
	SE2	0.801			
	SE3	0.810			
	SE4	0.819			
	SE5	0.830			
	SE6	0.777			
	SE7	0.790			
	SE8	0.821			
	SE9	0.831			
	SE10	0.827			
	SE11	0.861			
	SE12	0.848			
Self-reflection	SR1	0.762	0.650	0.918	0.917
	SR2	0.822			
	SR3	0.877			
	SR4	0.794			
	SR5	0.796			
	SR6	0.783			
Psychological resilience	PR1	0.748	0.708	0.960	0.961
	PR2	0.829			
	PR3	0.834			
	PR4	0.822			
	PR5	0.842			
	PR6	0.894			
	PR7	0.878			
	PR8	0.829			
	PR9	0.873			
	PR10	0.856			
Perceived organizational support	POS1	0.906	0.746	0.958	0.959
	POS2	0.926			
	POS3	0.925			
	POS4	0.888			
	POS5	0.795			
	POS6	0.738			
	POS7	0.856			
	POS8	0.845			
Job crafting	JC1	0.839	0.710	0.956	0.951
	JC2	0.890			
	JC3	0.906			
	JC4	0.900			
	JC5	0.840			
	JC6	0.837			
	JC7	0.893			
	JC8	0.877			
	JC9	0.537			

We considered but did not adopt the alternative of using the Job Crafting Self-Efficacy Scale (JCSES) developed by Roczniewska et al. (6). The JCSES measures employees' specific belief in their capability to perform job crafting behaviors. Using it as a predictor of job crafting would introduce construct proximity between predictor and outcome, thereby compromising the theoretical test of the general self-efficacy → job crafting pathway that this study is designed to examine. Prior studies examining this pathway, including Miraglia et al. (7) cited in our theoretical framework, have similarly relied on domain-general self-efficacy measures for this reason. All items were rated on a five-point Likert scale (1 = *not at all*, 5 = *very much*).

#### 3.1.2 Self-reflection scale

Self-reflection was measured using the self-reflection subscale of the Self-Reflection and Insight Scale (SRIS), consisting of 6 items that assess an individual's tendency to engage in reflection on their own thoughts, feelings, and behaviors (54). Each item was scored using a five-point Likert scale (1 = *strongly disagree*, 5 = *strongly agree*). This scale has been widely applied in organizational, educational, and clinical research with good reliability and validity.

#### 3.1.3 Psychological resilience scale

Psychological resilience was assessed using the 10-item Connor-Davidson Resilience Scale (CD-RISC-10) developed by Connor and Davidson (38). The CD-RISC-10 is a widely validated measure of general psychological resilience that captures individuals' capacity to maintain positive functioning, adapt effectively, and recover from setbacks when facing adversity, stress, and change (38). It has been extensively applied across occupational, clinical, and community populations with strong psychometric properties. Consistent with our theoretical framework, this study conceptualizes and measures resilience as a domain-general psychological resource, aligning the construct definition with the operational content of the CD-RISC-10 (11, 21). The scale uses a five-point rating format (1 = *never*, 5 = *always*), with higher scores indicating higher psychological resilience.

#### 3.1.4 Perceived organizational support scale

Perceived organizational support was assessed using an 8-item version of the Perceived Organizational Support Scale developed by Eisenberger et al. (55). Participants rated their perceived organizational support on a five-point Likert scale (1 = *strongly disagree*, 5 = *strongly agree*).

#### 3.1.5 Job crafting scale

Job crafting refers to employees' proactive adjustments to their tasks, relationships, and cognition to align with their personal needs and preferences. This study used a 9-item scale that has demonstrated good reliability and validity in the Chinese context, comprising three subdimensions: task crafting (3 items), relational crafting (3 items), and cognitive crafting (3 items). All items were rated on a five-point Likert scale (1 = *never*, 5 = *very frequently*).

#### 3.1.6 Translation procedure

All measurement scales used in this study were originally developed in English. To ensure linguistic and conceptual equivalence in the Chinese context, a standard translation and back-translation procedure was conducted (50, 51). The original items were translated into Chinese by two bilingual researchers, and the Chinese version was then independently back-translated into English by another bilingual researcher blind to the original items. Discrepancies were discussed and resolved through consensus to ensure semantic consistency and cultural appropriateness. Where cross-context adaptation was required (see Section 3.1.1), functionally equivalent referents were substituted while preserving item structure. Previous studies have successfully applied these scales in Chinese samples, supporting their contextual validity.

### 3.2 Ethical approval and informed consent

This study involved human participants: 576 adult female employees in Chinese tourism enterprises who voluntarily completed an anonymous electronic questionnaire. The study protocol was reviewed and approved by the Ethics Committee of the School of Tourism and Sports Health, Hezhou University, on January 5, 2025. All research procedures involving human participants were conducted in accordance with the ethical standards of the institutional research committee and with the Declaration of Helsinki and its later amendments. Because this study involved anonymous questionnaire data with minimal risk to participants, a formal approval number was not required under institutional guidelines; however, full ethics-committee review and approval were completed prior to data collection.

Informed consent was obtained from all participants prior to their participation. The consent process was conducted through a written electronic format embedded at the beginning of the online questionnaire. Participants were informed about the purpose of the study, the voluntary nature of participation, anonymity, confidentiality, and the use of data solely for academic research purposes. Only participants who explicitly indicated their consent electronically were allowed to proceed with the questionnaire.

### 3.3 Data collection procedure

For the purpose of this study, female knowledge workers in Chinese tourism enterprises are operationally defined as female employees in coordination, supervisory, and managerial roles whose work requires specialized service expertise, complex customer-facing judgment, and autonomous decision-making. This definition includes roles such as front-desk supervisors, ticketing and customer-relations coordinators, marketing and product-design professionals, and operations managers—positions in which service delivery, cross-functional coordination, and problem-solving depend substantially on tacit knowledge and interpretive judgment rather than routinized task execution. As will be shown in Section 4.1, 91.15% of the resulting sample holds supervisory or higher-level positions, which is consistent with this operational definition. The generalizability implications of this sample profile are discussed as a limitation in Section 5.5.

The data for this study were collected from female knowledge workers in Chinese tourism enterprises, including hotels, travel agencies, scenic area operators, and cultural performance companies. Two considerations motivated the selection of this population. First, women constitute a substantial majority of the workforce in China's tourism industry, particularly in front-desk reception, ticketing management, customer relations, and tourism product design—roles that form the operational backbone of the sector (16). Second, in service-intensive and highly interactive work environments, female employees are especially likely to face dual pressures from emotional labor and gendered role expectations, making their job crafting behaviors particularly relevant to examining dynamic relationships among self-efficacy, psychological resilience, and organizational support (17, 18).

Data collection followed a multi-stage sampling procedure combined with structured questionnaire distribution. In the first stage, the research team, in collaboration with the Guangxi Tourism Association and regional industry authorities, developed a sampling frame covering tourism enterprises across four Chinese provinces—Guangxi, Guangdong, Hunan, and Guizhou—selected to represent both economically developed coastal-adjacent regions and inland tourism-intensive regions. Within this frame, 38 tourism enterprises were approached, spanning the four sub-industries listed above (accommodation, travel services, scenic area operations, and cultural performance/leisure services). Of these, 32 enterprises (an enterprise-level participation rate of 84.2%) agreed to participate in the survey.

In the second stage, within each participating enterprise, stratified random sampling was applied based on job categories (front-desk reception, marketing and customer relations, operations management, and product/design roles) to ensure adequate representation across role types. Human resource departments at participating enterprises provided anonymized employee directories from which female employees meeting the inclusion criteria (female, holding a knowledge-work role as defined above, with at least 6 months of tenure at the current enterprise) were randomly sampled.

Prior to formal data collection, a pilot study was conducted with 30 female tourism employees to assess questionnaire comprehension, item clarity, and average completion time. Feedback from the pilot was used to refine item wording and to confirm face validity of the adapted self-efficacy items (see Section 3.1.1). The pilot data were not included in the main analysis.

Questionnaires were distributed in both paper and electronic formats between January and March 2025. All research assistants involved in distribution underwent standardized training covering questionnaire administration, confidentiality assurance, and data quality control. A total of 600 questionnaires were returned during the distribution period. During data screening, questionnaires with substantial missing responses or evident response patterns (e.g., identical answers across items, straight-lining) were excluded. As a result, 576 valid questionnaires were retained for subsequent analyses, yielding an effective response rate of 96%. Given the low proportion of missing data in the retained sample, listwise deletion was applied in all statistical analyses. This sample size substantially exceeds the minimum requirements for structural equation modeling and moderated mediation analysis (56).

### 3.4 Statistical analysis strategy

Data analysis was conducted using SPSS 26.0 and AMOS 26.0. Confirmatory factor analysis and covariance-based structural equation modeling were performed in AMOS 26.0 using maximum likelihood estimation on the 45 observed items grouped into five latent variables (SE, SR, PR, POS, JC). The alternative measurement model comparisons reported in Section 4.3 were computed using the same maximum likelihood estimation to ensure internal comparability across nested models.

Mediation and moderated mediation analyses were conducted using the PROCESS Macro v4.2 for SPSS (67) on composite mean scores of the five constructs, rather than on latent variables. This two-step analytical strategy—latent-variable CFA/SEM for measurement validation and composite-score PROCESS for path-level testing—is standard in applied job-crafting and organizational-behavior research and was chosen because PROCESS Model 14 does not natively support latent moderated mediation. For the parallel mediation model (H2 and H3), PROCESS Model 4 was used with self-efficacy as the predictor, self-reflection and psychological resilience as parallel mediators, and job crafting as the outcome. For the moderated mediation model (H4 and H5), PROCESS Model 14 was used, with perceived organizational support moderating both mediator-to-outcome paths (SR → JC and PR → JC). Age, marital status, education, work experience, and job title were entered as covariates in all regression and PROCESS models.

Bootstrap resampling with 10,000 iterations was used to construct bias-corrected 95% confidence intervals for indirect effects, conditional indirect effects, and moderated mediation indices. Variables were entered in raw (uncentered) form following PROCESS Model 14 defaults; interaction terms were computed as products of raw predictor and moderator. As a consequence of this specification, main-effect coefficients in the moderated regression represent conditional effects at the value of zero on the moderator scale rather than average effects; this is elaborated in Section 4.5.4. Multicollinearity across substantive predictors was assessed via variance inflation factors, tolerance values, and the condition index (reported in Section 4.5.1 and Supplementary Table S2b).

## 4 Results

### 4.1 Descriptive statistics

The 576 valid samples reveal typical characteristics of female knowledge workers in Chinese tourism enterprises (see Table 2). The sample is concentrated in the 30–49 age group (63.54%), reflecting employees in the critical mid-career stage in which they simultaneously navigate professional development, service-quality accountability, and family responsibilities. This age structure is consistent with women's role as the operational backbone of tourism enterprises. The majority of respondents are married (79.34%), which underscores the co-occurring work-family demands characteristic of gendered service labor in Chinese tourism contexts. Nearly 80% hold a bachelor's degree or higher, confirming the “knowledge worker” positioning of the sample. Regarding work experience, 40.63% of respondents have more than 20 years of tenure, while approximately one quarter have 1–5 years, indicating a dual structure of experienced senior personnel alongside a new-generation cohort. Managerial positions pre-dominate (supervisors 33.51%, department managers 30.38%, general managers 27.26%), suggesting that the sample primarily comprises women in coordination and decision-making roles who are structurally positioned to engage in task, relational, and cognitive crafting. Overall, the sample structure aligns closely with the study's theoretical focus on how self-efficacy, psychological resilience, self-reflection, and organizational support jointly relate to job crafting among female knowledge workers under the emotionally demanding and service-intensive conditions of Chinese tourism enterprises.

**Table 2:** Sample demographic information (*N* = 576).

Item	Category	Frequency	Percentage (%)	Cumulative (%)
Age	18–29	120	20.83	20.83
	30–39	177	30.73	51.56
	40–49	189	32.81	84.38
	Above 50	90	15.63	100.00
Marital status	Unmarried	98	17.01	17.01
	Married	457	79.34	96.35
	Divorced	21	3.65	100.00
Education	High school	4	0.69	0.69
	Vocational education	111	19.27	19.97
	Undergraduate	456	79.17	99.13
	Graduate	5	0.87	100.00
Work experience (years)	1–5	140	24.31	24.31
	6–10	134	23.26	47.57
	11–20	68	11.81	59.38
	Above 20	234	40.63	100.00
Job title	Supervisor	193	33.51	33.51
	Department manager	175	30.38	63.89
	General manager	157	27.26	91.15
	Employee	51	8.85	100.00

### 4.2 Common method bias test

Following procedural recommendations for reducing common method bias (57), several safeguards were implemented at the questionnaire design and administration stages: (a) full anonymity was assured, and participants were explicitly informed that their responses would be used solely for academic research to reduce social desirability and evaluation apprehension; (b) participants were told there were no right or wrong answers; (c) predictor, mediator, moderator, and criterion measures were placed in separate sections of the questionnaire and administered under different framing headings to reduce priming effects; and (d) the standard translation and back-translation procedure ensured item clarity to reduce ambiguity-driven response consistency. In addition to these procedural remedies, three statistical tests were conducted to evaluate the potential influence of common method bias *post hoc*.

First, Harman's single-factor test was performed via an exploratory factor analysis without rotation on all 45 measurement items (57). The first common factor before rotation explained 29.18% of the variance, well below the 40% critical threshold, and no single factor dominated the variance structure.

Second, a common latent factor (CLF) test was conducted in a confirmatory factor analysis framework by adding an unmeasured latent methods factor loading on all 45 items alongside the five theoretical latent factors. Model comparison indicated that adding the CLF improved fit (ΔCFI = +0.017), and the average CLF-attributable variance was 29.77%. Comparing standardized factor loadings on the theoretical factors before and after adding the CLF, the mean absolute change in loadings was 0.223. While this value is slightly above the 0.20 rule-of-thumb, three considerations argue against interpreting it as evidence that CMB drives the substantive findings. First, a pure common-method-variance mechanism would inflate all inter-construct correlations uniformly in the positive direction; it cannot generate a statistically significant negative interaction. The empirically observed PR × POS → JC interaction is significant and negative (β = −0.128, *p* = 0.001; see Section 4.5.3), which is theoretically incompatible with a CMB-driven artifact. Second, the two mediation pathways show substantially different magnitudes (PR: β = 0.354; SR: β = 0.141), a 2.5-fold difference that reflects genuine construct-level differentiation rather than uniform method inflation. Third, discriminant validity, tested via HTMT ratios and alternative measurement model comparisons (see Section 4.3), independently confirms that the five constructs are empirically distinguishable.

Third, the factorability of the data was verified. The Kaiser-Meyer-Olkin (KMO) measure of sampling adequacy was 0.974, and Bartlett's test of sphericity was significant (χ^2^ = 33,755.482, df = 1,176, *p* < 0.001), supporting the structural validity of the scales (58).

Taken together, these results indicate that CMB does not substantively account for the observed pattern of findings, though this issue is discussed further as a limitation in Section 5.5.

### 4.3 Reliability and validity

To ensure the reliability and validity of the research scales, confirmatory factor analysis (CFA) was conducted, and internal consistency reliability, convergent validity, and discriminant validity were examined (see Tables 1, 3, 4a).

**Table 3:** Discriminant validity test of latent variables (Fornell-Larcker).

Construct	SE	SR	PR	POS	JC
SE	**0.808**				
SR	0.425	**0.806**			
PR	0.650	0.453	**0.841**		
POS	0.413	0.662	0.481	**0.862**	
JC	0.634	0.544	0.706	0.550	**0.843**

Diagonal values (in bold) are the square roots of AVE. Off-diagonal values are inter-construct correlations.

**Table 4a:** Alternative measurement model comparison.

Model	χ^2^	df	Δχ^2^	Δdf	*p*	CFI	TLI	RMSEA
Five-factor (hypothesized)	3,893.67	935	—	—	—	0.894	0.888	0.074
Four-factor A (SR + PR combined)	5,946.67	939	2,053.00	4	<0.001	0.820	0.810	0.096
Four-factor B (SE + SR combined)	6,026.29	939	2,132.62	4	<0.001	0.817	0.807	0.097
Three-factor (SE + SR + PR combined)	8,583.78	942	4,690.11	7	<0.001	0.726	0.712	0.119
One-factor	13,888.76	945	9,995.09	10	<0.001	0.535	0.513	0.154

Fit indices for the alternative model comparison are computed under a consistent measurement model specification without correlated error terms; absolute values therefore differ from the fit indices in Table 4b. The HTMT matrix (all values < 0.85) is reported in Supplementary Table S2.

#### 4.3.1 Reliability

Cronbach's α coefficients and composite reliability (CR) values for all five latent variables exceeded 0.90, well above the 0.70 threshold recommended by Nunnally and Bernstein (52), indicating strong internal consistency (see Table 1).

#### 4.3.2 Convergent validity

Following Fornell and Larcker (53), convergent validity was assessed via average variance extracted (AVE) and standardized factor loadings. AVE values for self-efficacy (0.653), self-reflection (0.650), psychological resilience (0.708), perceived organizational support (0.746), and job crafting (0.710) all exceeded 0.50. Standardized factor loadings for all items were significant and exceeded 0.70, with the exception of two items (SE1 = 0.667 and JC9 = 0.537), which were retained given the strong overall AVE and CR values for their respective constructs. These results support convergent validity.

#### 4.3.3 Discriminant validity

Discriminant validity was assessed using two complementary criteria. First, the Fornell-Larcker criterion (53) requires that the square root of a latent variable's AVE exceed its correlations with other variables. As shown in Table 3, all constructs met this criterion. Second, given the relatively high inter-construct correlations (e.g., PR–JC = 0.706, SE–PR = 0.650), Heterotrait-Monotrait ratios (HTMT) were additionally computed as a more stringent test (59). All ten HTMT values fell below the conservative 0.85 threshold (maximum HTMT = 0.739 between PR and JC), confirming that the constructs are empirically distinguishable.

#### 4.3.4 Alternative measurement model comparisons

To further verify the distinctiveness of the five theoretical constructs, the hypothesized five-factor model was compared against four alternative models (see Table 4a): a four-factor model combining SR and PR; a four-factor model combining SE and SR; a three-factor model combining SE, SR, and PR; and a one-factor model. The hypothesized five-factor model demonstrated substantially better fit than all alternatives, with all Δχ^2^ tests significant at *p* < 0.001, providing strong evidence for the discriminant validity of the five-factor measurement structure.

### 4.4 Model fit

The overall fit of the structural model was tested, and the results indicate good fit (see Table 4b). The χ^2^/df ratio was 2.665 (< 3), and the RMSEA was 0.054 with 90% CI [0.051, 0.056], meeting the RMSEA ≤ 0.06 criterion (60, 66). The RMR (0.032) and SRMR (0.038) were both below 0.05, indicating low residuals. Incremental fit indices (CFI = 0.945, NFI = 0.915, NNFI = 0.941, IFI = 0.945, TLI = 0.941) all exceeded 0.90. GFI (0.836) and AGFI (0.815) fell slightly below 0.90, which is acceptable given the large sample size and model complexity (61). Parsimony-adjusted indices (PCFI = 0.874, PNFI = 0.846, PGFI = 0.739) all exceeded 0.50, suggesting a reasonable balance between fit and parsimony.

Table 4bModel fit indices for the structural model.

**Table:** 

Index	χ^2^	df	*p*	χ^2^/df	GFI	RMSEA	RMR	CFI	NFI	NNFI
Criterion	—	—	>0.05	<3	>0.90	<0.10	<0.05	>0.90	>0.90	>0.90
Value	2,438.641	915	0	2.665	0.836	0.054	0.032	0.945	0.915	0.941

**Table:** 

Index	TLI	AGFI	IFI	PGFI	PNFI	PCFI	SRMR	RMSEA 90% CI
Criterion	>0.90	>0.90	>0.90	>0.50	>0.50	>0.50	<0.10	—
Criterion	0.941	0.815	0.945	0.739	0.846	0.874	0.038	0.051–0.056

### 4.5 Hypothesis testing

#### 4.5.1 Direct effect test and multicollinearity check

Before regression analyses, multicollinearity across all substantive predictors was assessed. Variance inflation factor (VIF) values for self-efficacy (1.80), self-reflection (1.89), psychological resilience (1.93), and perceived organizational support (1.93) were all well below the conservative threshold of 5.0 (62), and corresponding Tolerance values (0.51–0.55) all exceeded 0.20. The maximum condition index was 24.55, below the 30 threshold. These diagnostics indicate that despite the relatively high inter-construct correlations reported in Table 3, multicollinearity is not a concern for the regression analyses.

Following the reviewer's request for greater clarity, the hierarchical regression previously presented in a single combined format is now reported in three separate tables (Tables 5a–c), each corresponding to a distinct dependent variable in the model.

**Table 5a:** Regression predicting job crafting (full moderated model).

Variable	β	SE	*t*	*p*	95% CI
Constant	−1.894	0.642	−2.949	0.003	—
SE	0.298	0.054	5.533	<0.001	(0.192, 0.404)
POS	0.370	0.162	2.284	0.023	—
SR	−0.044	0.087	−0.504	0.614	—
SR × POS	0.077	0.026	2.942	0.003	(0.010, 0.116)
PR	0.926	0.149	6.212	<0.001	—
PR × POS	−0.128	0.038	−3.394	<0.001	(−0.202, −0.054)
Age	0.027	0.051	0.533	0.595	—
Marriage	0.115	0.063	1.808	0.071	—
Education	−0.133	0.058	−2.301	0.022	—
Work year	0.016	0.040	0.409	0.683	—
Title	0.063	0.027	2.315	0.021	—

*R*^2^ = 0.642, Adjusted *R*^2^ = 0.635, *F*_(11,564)_ = 91.85, *p* < 0.001. Predictors are entered in raw (uncentered) form, following the standard PROCESS Model 14 specification. The conditional main effect of SR at POS = 0 is not significant; the effect of SR on JC as a function of POS is captured by the significant interaction term (see Section 4.5.4 for interpretation).

**Table 5b:** Regression predicting self-reflection.

Variable	β	SE	*t*	*p*
Constant	1.245	0.344	3.622	<0.001
SE	0.580	0.054	10.804	<0.001
Age	0.119	0.067	1.768	0.078
Marriage	0.025	0.084	0.300	0.765
Education	0.076	0.077	0.986	0.324
Work year	−0.114	0.053	−2.148	0.032
Title	0.074	0.036	2.049	0.041

*R*^2^ = 0.192, Adjusted *R*^2^ = 0.182, *F*_(6,569)_ = 22.47, *p* < 0.001.

**Table 5c:** Regression predicting psychological resilience.

Variable	β	SE	*t*	*p*
Constant	0.512	0.260	1.966	0.050
SE	0.790	0.041	19.448	<0.001
Age	0.140	0.051	2.753	0.006
Marriage	0.109	0.064	1.715	0.087
Education	−0.058	0.058	−0.992	0.321
Work year	−0.116	0.040	−2.895	0.004
Title	0.025	0.027	0.911	0.363

*R*^2^ = 0.440, Adjusted *R*^2^ = 0.434, *F*_(6,569)_ = 74.59, p < 0.001.

Table 5a reports the full moderated regression predicting job crafting. Self-efficacy shows a significant positive association with job crafting [β = 0.298, *p* < 0.001, 95% CI = (0.192, 0.404)], supporting H1. Table 5b shows that self-efficacy is significantly and positively associated with self-reflection (β = 0.580, *p* < 0.001), and Table 5c shows that self-efficacy is significantly and positively associated with psychological resilience (β = 0.790, *p* < 0.001). Together these establish the front-half paths of both mediation models.

#### 4.5.2 Mediation effect test

To test the mediating roles of self-reflection and psychological resilience (H2 and H3), bootstrap resampling (10,000 iterations) was used to compute bias-corrected 95% confidence intervals for the indirect effects (see Table 6).

**Table 6:** Bootstrap mediation results (10,000 iterations).

Path	Indirect effect (β)	Boot SE	95% CI	Significance
SE → SR → JC	0.141	0.038	(0.070, 0.223)	Significant
SE → PR → JC	0.354	0.057	(0.250, 0.473)	Significant

Self-reflection significantly mediates the relationship between self-efficacy and job crafting [indirect β = 0.141, 95% CI = (0.070, 0.223)], indicating that employees with higher self-efficacy are more likely to engage in reflective processing, which in turn enhances their job crafting behaviors. H2 is supported.

Psychological resilience also significantly mediates the relationship between self-efficacy and job crafting [indirect β = 0.354, 95% CI = (0.250, 0.473)], indicating that employees with higher self-efficacy demonstrate stronger adaptive capacity when facing challenges, which is in turn associated with job crafting. H3 is supported.

Comparison of the two indirect effects reveals that the mediation effect through psychological resilience (β = 0.354) is substantially stronger than that through self-reflection (β = 0.141)—approximately 2.5 times larger. This pattern suggests that, for female knowledge workers in Chinese tourism enterprises, the adaptive pathway (psychological resilience) plays a more prominent role than the cognitive pathway (self-reflection) in translating self-efficacy into proactive job crafting behavior. This asymmetry is interpreted theoretically in Section 5.2.

#### 4.5.3 Moderation effect test

To examine the boundary role of POS in the resource conversion mechanism, PROCESS Model 14 was used to test the moderating effects of POS on both the SR → JC and PR → JC pathways, and to compute the moderated mediation indices (see Tables 7, 8).

**Table 7:** Moderation analysis results.

Path	β	SE	*t*	*p*	95% CI
SR × POS → JC	0.077	0.033	2.315	0.021	(0.010, 0.116)
PR × POS → JC	−0.128	0.033	−3.394	0.001	(−0.202, −0.054)

**Table 8:** Index of moderated mediation.

Path	Index	Boot SE	95% CI	Significance
SE → SR → JC (by POS)	0.045	0.022	(−0.001, 0.083)	Not significant
SE → PR → JC (by POS)	−0.101	0.039	(−0.174, −0.016)	Significant

##### 4.5.3.1 H4 (POS moderates SR → JC)

**Figure 2:**
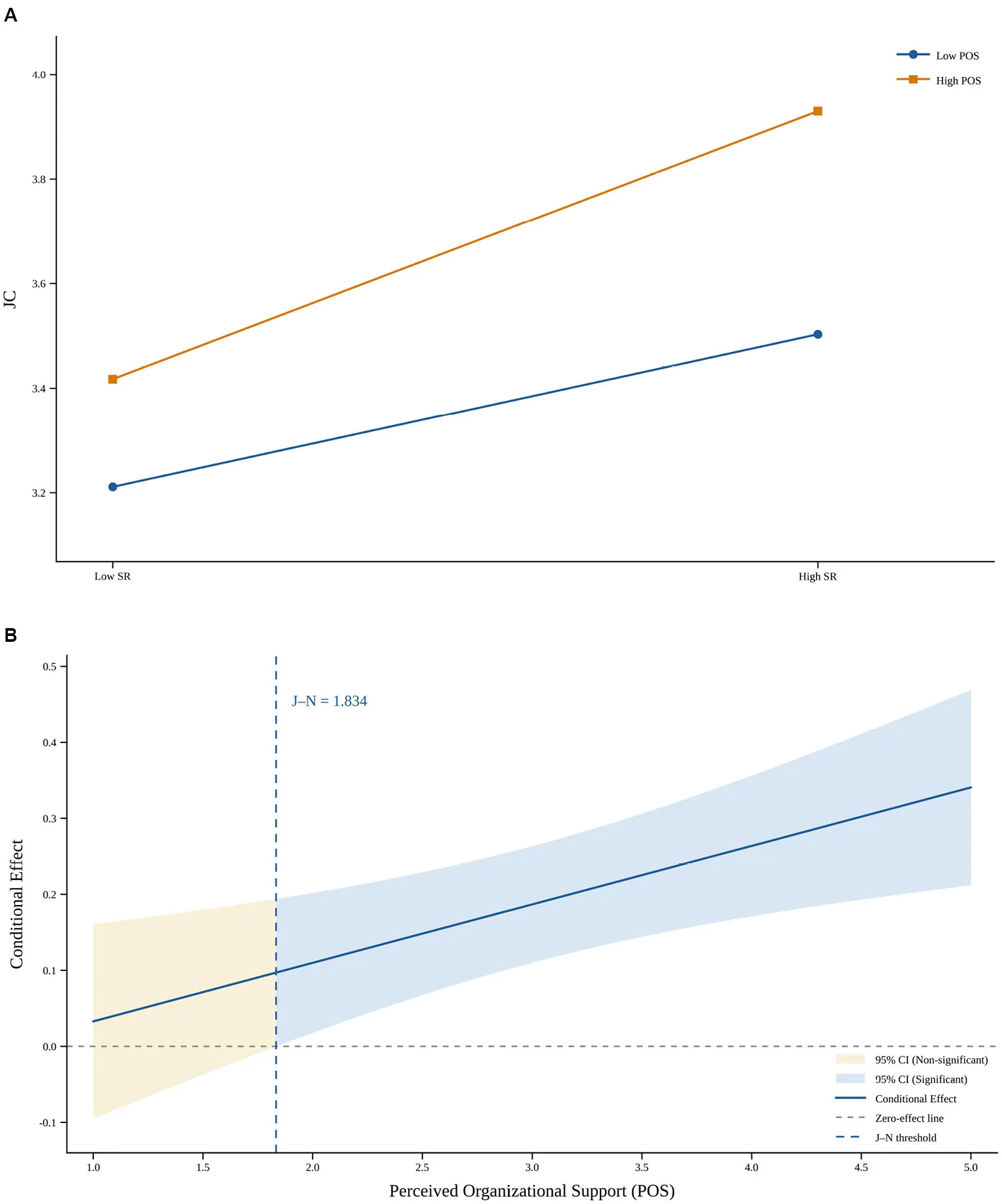
The moderating effect of POS on the relationship between SR and JC (simple slopes and Johnson-Neyman probing). **(A)** Simple slopes; **(B)** Johnson-Neyman plot.

The interaction term SR × POS is significantly and positively associated with JC [β = 0.077, *p* = 0.021, 95% CI = (0.010, 0.116)]. Simple slope analysis and Johnson-Neyman probing (Figure 2) show that the positive effect of self-reflection on job crafting is stronger when POS is higher and weaker when POS is lower. H4 is supported. This result indicates that POS functions as an amplifier of the cognitive resource pathway: contextual support enables employees to translate reflective insights into behavioral action, consistent with the resource complementarity logic of COR theory.

##### 4.5.3.2 H5 (POS moderates PR → JC)

**Figure 3:**
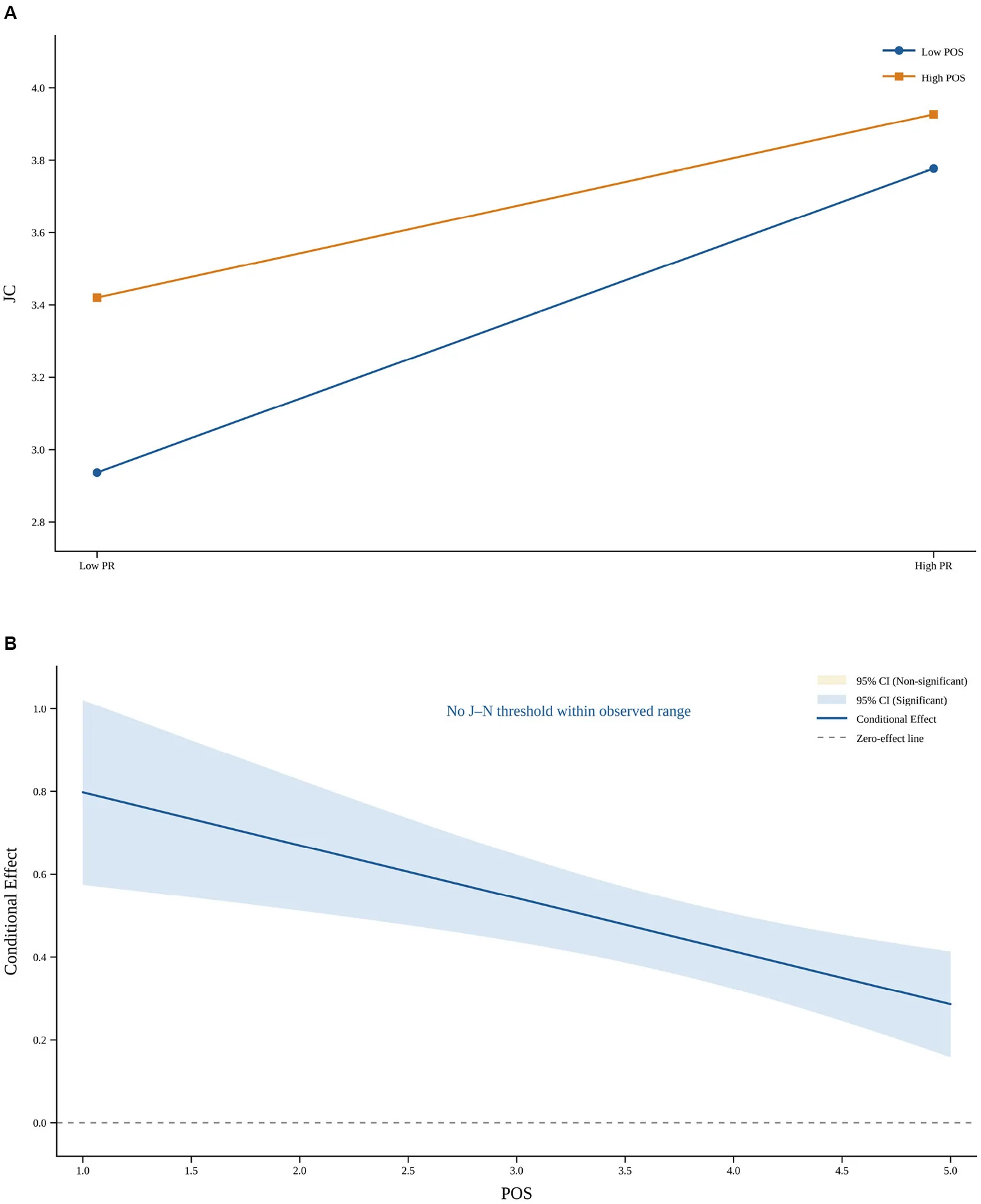
The moderating effect of POS on the relationship between PR and JC (simple slopes and Johnson-Neyman probing). **(A)** Simple slopes; **(B)** Johnson-Neyman plot.

H5 predicted that POS would positively moderate the psychological resilience–job crafting relationship. Contrary to this prediction, the interaction term PR × POS is significantly negative [β = −0.128, *p* = 0.001, 95% CI = (−0.202, −0.054)]. Simple slope analysis and Johnson-Neyman probing (Figure 3) show that the positive effect of psychological resilience on job crafting is stronger when POS is lower, and weakens as POS increases. H5 is therefore not supported in the predicted direction. Instead, this significant negative interaction reveals a resource substitution effect: when contextual resources (POS) are abundant, employees' reliance on their internal adaptive resource (psychological resilience) is reduced; when contextual resources are scarce, psychological resilience becomes a more essential internal substitute for external support. The theoretical significance of this finding is elaborated in Section 5.2.

##### 4.5.3.3 Moderated mediation

The moderated mediation index for SE → SR → JC via POS is not significant [Index = 0.045, 95% CI = (−0.001, 0.083)], indicating that while H4 holds at the interaction level, the full conditional indirect pathway does not reach statistical significance across the observed POS range. In contrast, the moderated mediation index for SE → PR → JC via POS is significant and negative [Index = −0.101, 95% CI = (−0.174, −0.016)], confirming that the resource substitution effect propagates through the full mediated pathway.

Together, these results reveal that POS operates asymmetrically across the two resource pathways: it amplifies the cognitive (reflection) pathway while substituting for the adaptive (resilience) pathway. This asymmetry represents a boundary condition of COR theory that has not been previously articulated in the job crafting literature and constitutes one of the central theoretical contributions of this study.

#### 4.5.4 Interpretive note on the coefficient of SR

A brief interpretive note is warranted regarding the coefficient of SR in Table 5a. In the full moderated model, the main-effect coefficient of SR appears non-significant (β = −0.044, *p* = 0.614). This is the conditional coefficient at POS = 0 in the raw (uncentered) specification used by PROCESS Model 14; it does not represent the average effect of SR on JC. The average (unconditional) indirect effect through SR is significant (β = 0.141; see Table 6), and the interaction between SR and POS is significant (β = 0.077; see Table 7). These coefficients therefore answer different theoretical questions: the coefficient in Table 5A captures the effect at a single (out-of-range) level of POS, the coefficient in Table 6 captures the average indirect effect over the observed range of POS, and the coefficient in Table 7 captures how the SR–JC slope varies with POS. Their coexistence reflects the standard structure of conditional process analysis rather than a statistical inconsistency.

## 5 Discussion

### 5.1 Summary of key findings

Grounded in COR theory, this study investigated how personal and contextual resources jointly relate to job crafting among female knowledge workers in Chinese tourism enterprises. Using structural equation modeling and moderated mediation analysis, the results reveal four key findings. First, self-efficacy shows a significant positive association with job crafting. Second, this relationship is transmitted through two parallel psychological mechanisms—self-reflection and psychological resilience—confirming that self-efficacy is not directly linked to proactive behavior but operates through distinct cognitive and adaptive pathways. Notably, the adaptive pathway (psychological resilience) transmits a substantially stronger indirect effect than the cognitive pathway (self-reflection). Third, POS amplifies the effect of self-reflection on job crafting, supporting the resource complementarity logic within the cognitive pathway. Fourth, and contrary to the original prediction, POS weakens the effect of psychological resilience on job crafting, revealing an unexpected but theoretically significant resource substitution effect. Together, these findings expose an asymmetric structure of resource conversion in service-intensive tourism contexts: contextual resources do not uniformly enhance internal resources but interact with them in pathway-specific and functionally differentiated ways.

### 5.2 Comparison with existing literature

The positive association between self-efficacy and job crafting identified in this study is consistent with a substantial body of prior research conceptualizing self-efficacy as a core personal resource motivating proactive work behaviors (3, 6, 7). From a COR perspective, employees with stronger efficacy beliefs are more willing to invest personal resources in proactive actions because they expect such investments to yield future resource gains (11, 12). The present findings further confirm that this mechanism is particularly salient in service-intensive tourism contexts, where employees face high emotional labor and situational uncertainty (1).

Beyond confirming the main effect, this study extends existing literature by demonstrating that the influence of self-efficacy on job crafting operates through differentiated psychological pathways. While prior studies have often examined single mediators—such as motivation, engagement, or resilience—in isolation (4, 10), the current findings show that self-reflection and psychological resilience function as parallel but distinct mechanisms. Importantly, the two pathways are not equivalent in magnitude: the indirect effect through psychological resilience is approximately 2.5 times larger than that through self-reflection. This asymmetry suggests that, for female knowledge workers in Chinese tourism enterprises, adaptive capacity is a more proximal correlate of proactive job redesign than reflective cognition. A plausible interpretation is that tourism knowledge work is characterized by high situational unpredictability, emotional labor intensity, and demand fluctuation, all of which reward adaptive resource mobilization more strongly than deliberate cognitive processing. In such contexts, the capacity to sustain functioning under stress may be associated with observable job crafting behaviors more directly than reflection-based sense-making, which requires cognitive bandwidth that is often scarce during high-tempo service encounters. This finding refines the mixed evidence in earlier research regarding which internal mechanism best explains the self-efficacy–job crafting link (3, 7) and highlights the need to differentiate cognitive vs. adaptive resource pathways when theorizing about proactive behavior.

The mediating role of self-reflection nevertheless aligns with studies emphasizing reflective cognition as a correlate of learning, adaptation, and proactive change (1, 20). In contrast to research that treats reflection as a peripheral or *post-hoc* process, this study positions self-reflection as a cognitive bridge that translates efficacy beliefs into concrete job crafting behaviors—though its role, in this population, appears to be complementary to rather than dominant over the adaptive mechanism.

The mediating role of psychological resilience is consistent with prior work highlighting resilience as a critical psychological resource for maintaining proactive functioning under pressure and uncertainty (6, 10, 64, 65). By empirically linking psychological resilience to job crafting in a tourism enterprise sample, the findings provide additional support for the resource gain spiral proposed in COR theory, whereby personal resources reinforce one another to sustain proactive behavior over time (12).

With regard to contextual influences, the moderating role of POS partially corroborates earlier studies suggesting that supportive organizational environments facilitate proactive behavior by reducing perceived risks and enhancing psychological safety (8, 13). However, this study departs from the dominant assumption that POS is uniformly beneficial. The finding that POS weakens the psychological resilience–job crafting relationship reveals a resource substitution effect that has not been previously articulated in the job crafting literature. From a COR perspective, this pattern is theoretically coherent: contextual resources and internal adaptive resources can address overlapping functional demands (buffering stress, absorbing risk, sustaining functioning under pressure), and when contextual support is abundant, the marginal utility of internal adaptive capacity for driving proactive behavior diminishes. Conversely, in low-POS contexts, employees must draw more heavily on their own psychological resilience to enact job crafting, making the resilience–behavior link stronger. This asymmetric pattern—POS amplifying the cognitive pathway while substituting for the adaptive pathway—suggests that the moderating role of contextual resources depends on the functional overlap between contextual and internal resources within a specific psychological pathway. When contextual resources supply what the internal resource cannot (e.g., organizational sanction for reflection-derived action), they amplify; when they duplicate what the internal resource already provides (e.g., stress buffering, adaptive functioning), they substitute. This nuanced result extends prior POS research (13, 63) and offers an important refinement of COR theory by demonstrating that the effects of contextual resources depend on the specific psychological pathway through which behavior is enacted.

### 5.3 Theoretical implications

This study makes three theoretical contributions. First, it advances job crafting research by proposing and empirically testing a parallel mediation model in which self-efficacy is related to job crafting through both cognitive (self-reflection) and adaptive (psychological resilience) pathways (3, 7). Empirical comparison of the two pathways further reveals that the adaptive pathway carries substantially greater weight than the cognitive pathway in this population, providing a more precise account of how efficacy beliefs relate to proactive behavior in service-intensive contexts. This dual-path perspective refines existing linear models and responds to calls for greater theoretical precision in explaining resource-conversion processes (1, 9).

Second, and most significantly, the study contributes to COR theory by revealing an asymmetric structure of contextual resource effects. By demonstrating that POS amplifies reflection-driven job crafting while substituting for resilience-driven job crafting, the findings provide empirical boundary conditions for the resource gain spiral proposition and introduce a pathway-specific formulation of the amplification-vs.-substitution distinction. This refines the widespread but untested assumption that contextual resources uniformly enhance personal-resource effects on proactive behavior (13, 63). Whether POS amplifies or substitutes appears to depend on the functional overlap between contextual and internal resources: amplification occurs where the two resources are functionally complementary (as with reflection-derived cognition requiring organizational sanction for behavioral enactment), and substitution occurs where the two resources are functionally overlapping (as with resilience and organizational buffering both serving adaptive/stress-mitigation functions). This distinction opens a new theoretical direction for COR research and calls for reconsideration of how contextual resources are theorized in relation to specific psychological mechanisms.

Third, by focusing on female knowledge workers in Chinese tourism enterprises, this study contextualizes resource conversion within a service-intensive and gendered work environment (16, 18). It demonstrates that adaptive mechanisms are especially salient for employees facing high emotional labor, seasonal demand fluctuation, and gendered role expectations, thereby extending job crafting theory to a population and context that have received limited attention in prior research (17, 24).

### 5.4 Practical and public health implications

First, tourism enterprises should actively enhance employees' self-efficacy as a foundation for job crafting (7). Hotels and travel agencies may introduce mentoring or buddy programs in which experienced employees guide newcomers through complex service scenarios, helping them build efficacy through mastery experiences (8, 10).

Second, managers should institutionalize reflection mechanisms to strengthen the cognitive pathway to job crafting (20). Concrete practices may include post-service debriefings, structured customer case discussions, or reflection logs that help employees translate service experiences into improvement strategies (4, 33). Given that POS was found to amplify the reflection-driven pathway, organizations should pair reflection practices with clear signals of psychological safety and organizational endorsement of employee-initiated changes—managers should visibly welcome, protect, and act upon reflection-derived suggestions.

Third, and importantly, the resource substitution effect identified in this study cautions against assuming that “more POS is always better.” When organizational support becomes abundant, employees may become less reliant on their own psychological resilience, potentially undermining the very internal capacity that drives sustained proactive behavior. From a public health perspective, this finding has significant implications for employee mental health and occupational wellbeing in service-intensive sectors. Tourism enterprises should therefore support psychological resilience without creating overdependence: providing emotional support and buffering resources when genuinely needed, while simultaneously encouraging autonomy, problem-solving initiative, and self-directed recovery from setbacks (21, 22, 43). For instance, rather than immediately intervening in service failures, supervisors can guide employees to develop their own solutions, thereby preserving resilience-driven proactivity and safeguarding the psychological resources that protect against emotional exhaustion and burnout in service labor.

Fourth, given the high proportion of female employees in tourism, firms should adopt female-friendly human resource policies, such as flexible scheduling, career development programs, and inclusive leadership practices (16, 18). These measures can support internal resource development while ensuring that organizational support complements rather than replaces employees' proactive capacities (17). Such policies are also aligned with public health efforts to address occupational stress, psychosocial risks, and mental health inequalities among female workers in emotionally demanding service industries.

### 5.5 Limitations and avenues for future research

Several limitations should be acknowledged, which also point to promising directions for future research.

First, this study adopts a cross-sectional design, with data collected at a single point in time. Consequently, all findings reported in this study should be interpreted as associations consistent with the proposed theoretical model rather than as evidence of causal effects. Structural equation modeling and PROCESS-based analyses can evaluate whether the observed data are consistent with the specified theoretical pathways, but they cannot establish temporal precedence or rule out reverse causation or omitted-variable confounding. Future research could adopt longitudinal or experimental designs to capture the dynamic evolution of resource conversion processes. Collecting data across different stages of the tourism cycle (e.g., peak vs. off-peak seasons) would allow examination of whether the relative importance of cognitive reflection and psychological resilience shifts under varying environmental pressure, providing a stronger test of the resource gain and resource substitution mechanisms.

Second, common method bias, while systematically evaluated through both Harman's single-factor test and a CFA-based common latent factor test, cannot be entirely ruled out in cross-sectional survey research. Although convergent theoretical, empirical, and diagnostic evidence (particularly the significant negative interaction and the differentiated magnitudes of the two indirect effects) argues against a CMB-driven interpretation, future research would benefit from multi-source or time-lagged designs that reduce common method concerns at the design stage.

Third, the self-efficacy measure used in this study was contextually adapted from an instrument originally developed for the teaching context. Although we systematically adapted item referents, pre-tested the adapted items, and demonstrated strong psychometric properties in the current sample, the development of a fully validated tourism-specific self-efficacy scale remains an important direction for future research. Such an instrument could more precisely capture efficacy beliefs tied to emotional labor management, customer relationship co-creation, and service innovation.

Fourth, this study measures resilience using the CD-RISC-10, which captures general psychological resilience. Future research could complement this measure with career-specific or task-specific resilience measures to examine whether the mediation and substitution effects observed here vary across resilience types.

Fifth, the scope of variables included in this study is necessarily limited. Future research could extend the framework by incorporating additional organizational and team-level variables. Inclusive leadership may function as a critical contextual resource by fostering psychological safety and encouraging reflective behaviors, thereby strengthening the cognitive pathway. Team climate (e.g., supportive or innovative climate) may shape how individual resources are associated with proactive work at the collective level. Multi-level models could examine how leadership and team contexts interact with individual resources to influence job crafting.

Sixth, the sample is pre-dominantly composed of supervisory and managerial female employees (91.15% supervisors, department managers, or general managers), which aligns with our operational definition of female knowledge workers in tourism enterprises (see Section 3.3) but nevertheless limits generalizability to frontline non-managerial staff. Such staff may occupy different positions in the resource-conversion process—for example, having lower autonomy over task boundaries, more standardized service scripts, and different exposure to organizational support signals—which could alter the observed pathways. Future research should include stratified samples across managerial and non-managerial female employees to test whether the cognitive and adaptive pathways operate similarly across levels of the organizational hierarchy.

Seventh, this study focuses exclusively on female knowledge workers in Chinese tourism enterprises, which may further limit the generalizability of the findings. Future research could extend the model to mixed-gender samples or conduct cross-cultural comparisons to explore whether gender and cultural context moderate the resource-to-job-crafting process, particularly the boundary conditions of the substitution effect identified here.

## 6 Conclusion

Grounded in Conservation of Resources theory, this study advances the understanding of job crafting among female knowledge workers in Chinese tourism enterprises by demonstrating that self-efficacy is associated with proactive job redesign through two parallel psychological pathways—self-reflection and psychological resilience—with the adaptive pathway substantially outweighing the cognitive pathway in magnitude. More consequentially, this study reveals that perceived organizational support operates asymmetrically across these pathways, amplifying the cognitive pathway while substituting for the adaptive pathway. This asymmetric structure identifies a previously untheorized boundary condition of the resource gain spiral and cautions against the widespread assumption that contextual resources uniformly enhance internal-resource effects. For tourism enterprises and public health practitioners concerned with the occupational wellbeing of female service workers, the findings suggest that supporting proactive job redesign requires not only providing organizational support but also carefully preserving employees' internal resource capacity—particularly their psychological resilience—as an independent foundation for sustained proactive behavior.

## Data Availability

The original contributions presented in the study are included in the article/Supplementary material, further inquiries can be directed to the corresponding author.
