# Supplementary material for: Cognitive and adaptive pathways linking self-efficacy and job crafting: the moderating role of perceived organizational support among female knowledge workers in Chinese tourism enterprises
Source: Front Public Health. 2026 Aug 12;14:1886029. doi: 10.3389/fpubh.2026.1886029 (PMC13506892; doi:10.3389/fpubh.2026.1886029)
Supplement: Supplementary file 1 [file Table_1.docx]

# Supplementary Material

## Supplementary Table S1. Contextual Adaptation of the Self-Efficacy Scale Items

The following table presents the original teacher self-efficacy items from Tschannen-Moran and Hoy (1), the systematically adapted items used in the present study, and the Chinese back-translated version administered to female knowledge workers in tourism enterprises. Adaptation preserved the underlying capability structure (management, motivation, cooperation) while replacing educational referents with functionally equivalent tourism work referents, following the standard translation and back-translation procedure (2, 3).

| Item | Original Item (Tschannen-Moran & Hoy, 2001) | Adapted Item (English, Present Study) | Adapted Item (Chinese, Present Study) |
| --- | --- | --- | --- |
| SE1 | How much can you do to get through to the most difficult students? | How capable are you of engaging colleagues or customers who show low motivation? | 你有多大能力去调动那些积极性较低的同事或顾客？ |
| SE2 | How much can you do to help your students think critically? | How capable are you of motivating colleagues who show low engagement in their work? | 你有多大能力激励那些工作投入度较低的同事？ |
| SE3 | How much can you do to control disruptive behavior in the classroom? | How capable are you of getting colleagues to believe they can perform well at work? | 你有多大能力让同事相信他们能够胜任工作？ |
| SE4 | How much can you do to motivate students who show low interest in schoolwork? | How capable are you of helping colleagues value their work? | 你有多大能力帮助同事认识到自身工作的价值？ |
| SE5 | To what extent can you make your expectations clear about student behavior? | How capable are you of helping colleagues think critically about work problems? | 你有多大能力帮助同事批判性地思考工作中的问题？ |
| SE6 | How much can you do to get students to believe they can do well in schoolwork? | How capable are you of responding to difficult questions from customers or colleagues? | 你有多大能力回应来自顾客或同事的棘手问题？ |
| SE7 | How well can you respond to difficult questions from your students? | How capable are you of assessing whether team members understand shared work plans? | 你有多大能力判断团队成员是否理解共同的工作方案？ |
| SE8 | How well can you establish routines to keep activities running smoothly? | How capable are you of formulating effective questions to clarify work tasks? | 你有多大能力提出有效的问题来澄清工作任务？ |
| SE9 | How much can you do to help your students value learning? | How capable are you of handling disruptive incidents at work? | 你有多大能力处理工作中突发的干扰性事件？ |
| SE10 | How much can you gauge student comprehension of what you have taught? | How capable are you of clearly communicating expectations about workplace behavior? | 你有多大能力清晰地传达对工作行为的期望？ |
| SE11 | To what extent can you craft good questions for your students? | How capable are you of establishing routines that keep work activities running smoothly? | 你有多大能力建立起让工作有序运转的常规流程？ |
| SE12 | How much can you do to foster student creativity? | How capable are you of getting team members to follow workplace rules and procedures? | 你有多大能力促使团队成员遵守工作规范与流程？ |

*Note. The adaptation preserved the underlying three-dimensional structure of the original scale (efficacy for management, efficacy for motivation, efficacy for cooperation), which represent generalizable workplace capability domains rather than teaching-exclusive competencies. Adaptation was completed prior to data collection through the standard translation and back-translation procedure (2, 3). A pilot study with 30 female tourism employees confirmed the face validity and comprehension of the adapted items. Confirmatory factor analysis in the main sample (N = 576) yielded strong psychometric properties: standardized factor loadings ranged from 0.667 to 0.861, AVE = 0.653, CR = 0.957, and Cronbach’s α = 0.959.*

## Supplementary Table S2a. Heterotrait-Monotrait Ratios (HTMT) for Discriminant Validity

|  | SE | SR | PR | POS | JC |
| --- | --- | --- | --- | --- | --- |
| SE | — |  |  |  |  |
| SR | 0.453 | — |  |  |  |
| PR | 0.677 | 0.478 | — |  |  |
| POS | 0.429 | 0.702 | 0.502 | — |  |
| JC | 0.667 | 0.574 | 0.739 | 0.574 | — |

*Note. All HTMT values fall below the strict threshold of 0.85 (4), confirming discriminant validity across all five constructs. The maximum value is HTMT(PR, JC) = 0.739.*

## Supplementary Table S2b. Multicollinearity Diagnostics

| Predictor | VIF | Tolerance |
| --- | --- | --- |
| Self-Efficacy (SE) | 1.80 | 0.555 |
| Self-Reflection (SR) | 1.89 | 0.529 |
| Psychological Resilience (PR) | 1.93 | 0.518 |
| Perceived Organizational Support (POS) | 1.93 | 0.519 |
| Age | 4.65 | 0.215 |
| Marriage | 1.39 | 0.718 |
| Education | 1.16 | 0.862 |
| Work Year | 4.44 | 0.225 |
| Title | 1.27 | 0.785 |

*Note. The maximum condition index across the primary predictor set is 24.55, below the 30 threshold. All VIF values for substantive predictors are well below the conservative 5.0 threshold (5). The elevated VIFs for Age and Work Year reflect their expected correlation and do not affect interpretation of the primary predictors.*

## Supplementary Table S2c. Descriptive Statistics of Latent Variables

| Construct | k Items | M | SD | Skewness | Kurtosis | Split-Half r |
| --- | --- | --- | --- | --- | --- | --- |
| Self-Efficacy (SE) | 12 | 4.108 | 0.606 | -0.234 | -0.389 | 0.927 |
| Self-Reflection (SR) | 6 | 4.028 | 0.827 | -1.161 | 2.094 | 0.893 |
| Psychological Resilience (PR) | 10 | 3.885 | 0.752 | -0.159 | -0.444 | 0.943 |
| Perceived Organizational Support (POS) | 8 | 3.734 | 0.865 | -0.715 | 0.882 | 0.934 |
| Job Crafting (JC) | 9 | 3.512 | 0.924 | -0.417 | -0.278 | 0.911 |

*Note. All univariate distributions are within acceptable normality ranges for maximum likelihood estimation. Split-half reliability coefficients (Spearman-Brown corrected) further support internal consistency.*

## Supplementary Table S2d. Common Latent Factor (CLF) Test Summary

The CLF test was implemented within a CFA framework by adding an unmeasured latent methods factor loading on all 45 items alongside the five theoretical latent factors, with the CLF constrained to be orthogonal to the theoretical constructs.

| Statistic | Value |
| --- | --- |
| Baseline 5-factor CFA: χ² (df) | 3893.67 (935) |
| Baseline 5-factor CFA: CFI / TLI / RMSEA | 0.894 / 0.888 / 0.074 |
| 5-factor CFA with CLF: χ² (df) | 3371.69 (890) |
| 5-factor CFA with CLF: CFI / TLI / RMSEA | 0.911 / 0.901 / 0.070 |
| Mean absolute change in standardized loadings (baseline vs. with CLF) | 0.223 |
| Mean CLF-attributable variance | 29.77% |

*Note. Although the mean loading change is marginally above the 0.20 rule of thumb, the significant negative interaction term (PR × POS → JC, β = -0.128, p = 0.001) and the differentiated magnitudes of the two indirect effects (PR: 0.354; SR: 0.141) provide theoretical evidence against a common-method-variance interpretation, as CMB inflates covariances uniformly in the positive direction. Discriminant validity is independently supported by HTMT ratios (all < 0.85; see Supplementary Table S2a) and alternative measurement model comparisons (see Table 4a in the main manuscript).*

## References for Supplementary Material

1. Tschannen-Moran M, Hoy AW. Teacher efficacy: capturing an elusive construct. *Teach Teach Educ* (2001) 17:783–805. doi: 10.1016/S0742-051X(01)00036-1
2. Brislin RW. Back-translation for cross-cultural research. *J Cross Cult Psychol* (1970) 1:185–216. doi: 10.1177/135910457000100301
3. Beaton DE, Bombardier C, Guillemin F, Ferraz MB. Guidelines for the process of cross-cultural adaptation of self-report measures. *Spine* (2000) 25:3186–91. doi: 10.1097/00007632-200012150-00014
4. Henseler J, Ringle CM, Sarstedt M. A new criterion for assessing discriminant validity in variance-based structural equation modeling. *J Acad Mark Sci* (2015) 43:115–35. doi: 10.1007/s11747-014-0403-8
5. Hair JF, Risher JJ, Sarstedt M, Ringle CM. When to use and how to report the results of PLS-SEM. *Eur Bus Rev* (2019) 31:2–24. doi: 10.1108/EBR-11-2018-0203
